# Lifestyle Strategies for the Management of Obesity in Older Adults: From Controversies to Alternative Interventions

**DOI:** 10.3390/healthcare10102107

**Published:** 2022-10-21

**Authors:** Anthony Villani

**Affiliations:** School of Health and Behavioural Sciences, University of the Sunshine Coast, Sippy Downs, QLD 4556, Australia; avillani@usc.edu.au

Improvements in infection control, management of chronic diseases and impressive advancements in modern medicine have all contributed to increases in life expectancy [1,2]. Nevertheless, despite population ageing, evidence that increased longevity is accompanied by healthy ageing is indeed scant [2]. A number of age-related chronic diseases are mediated by overweight and obesity [3]. Obesity (when measured by BMI) is a well-established risk factor for chronic disease and pre-mature mortality in adult populations [4,5,6]. However, the interpretation of BMI classifications and diagnostic cut-points, particularly in an older adult population, remains controversial. Specifically, a large meta-analysis (~200,000 individuals aged ≥65 years) reported a U-shaped relationship between BMI and mortality risk, with the lowest risk represented by a BMI between 24 and 30 kg/m^2^ [7]. However, the so-called ‘obesity paradox’ can at least be partially explained by the marked heterogeneity (e.g., study population, residual confounders, length of follow-up, etc.) across studies, supporting the presence of this phenomenon [8]. Perhaps most pertinently, BMI is also not a direct measure of adiposity [9]. Nevertheless, secular trends in obesity amongst the older adult population have steadily increased over recent decades, making this an important public health priority [10,11]. 

A number of hallmark physiological changes are associated with ageing, including reductions in lean body mass and a concurrent accumulation of fat mass [11]. Of greater concern, however, is the redistribution of subcutaneous adipose tissue to the abdominal visceral compartment [12]. Excessive accumulation of visceral fat is a well-established risk factor for cardiometabolic disorders, including cardiovascular disease, metabolic syndrome, and type 2 diabetes. Moreover, an age-related decline in skeletal muscle mass and function is termed sarcopenia [13], which is hereafter classified as a disease (‘muscle failure’, ICD-10-CM M62.84) grounded in adverse changes to skeletal muscle architecture that accrue across the lifespan. The interplay between sarcopenia and excess adiposity, a phenomenon termed sarcopenic obesity, has recently emerged as an important public health concern in older populations. As such, there is mounting evidence to suggest that the co-existence of both sarcopenia and obesity increase the risk of adverse cardiometabolic and functional impairments compared with either condition alone [10,14]. Therefore, effective interventions are required to promote cardiometabolic and musculoskeletal health in overweight and obese older adults.

Nevertheless, the presentation of overweight and obesity in older adults presents a complex challenge to healthcare professionals for the prescription of appropriate interventions. Whilst weight loss is likely to result in favorable cardiometabolic outcomes, one such challenge and source of controversy is the potential negative consequences associated with energy restricted diets on lean body mass, bone mineral density, and functional status [15,16]. Exercise prescription and nutrition are two important lifestyle interventions to facilitate optimal changes in body composition with age. The addition of resistance training to an energy-restricted diet has previously been shown to attenuate the loss of lean body mass, increase strength and ameliorate frailty in obese older adults [17,18,19]. Furthermore, resistance training independently improves cardiometabolic health [20] and ameliorates low-grade inflammation in the elderly [21,22]. Nevertheless, Colleluori et al. [23] previously showed that aerobic exercise combined with resistance training was more effective than aerobic or resistance training alone in improving muscle protein synthesis and myocellular quality during intentional energy-restricted weight loss in obese older adults.

With regards to nutrition, manipulating dietary composition during intentional energy intake restriction, particularly dietary protein, may attenuate the loss of lean body mass [24]. Dietary protein intake stimulates muscle protein synthesis and facilitates postprandial muscle protein accretion [25]. As such, adequate intake of dietary protein is imperative, particularly given that the elderly are less able to efficiently utilize amino acids for muscle protein synthesis, resulting in a blunted muscle protein synthetic response to an anabolic stimuli, a phenomenon termed anabolic resistance [25]. Undoubtably, dietary protein requirements increase with age, which is a topic of conversation established by several expert groups [26,27,28]. Recommended protein intakes for otherwise healthy adults vary worldwide but are generally established between 0.8 and 1.0 g protein/kg body weight (BW)/day. Nevertheless, expert and consensus groups have advocated that dietary protein recommendations should be set to at least 1.0–1.2 g/kg BW/day, or even greater, depending on the health and nutritional status, body composition, and physical activity levels of older adults [26,27,28,29]. Furthermore, to account for the potential blunted muscle protein synthetic response to the anabolic stimuli of dietary protein in response to ageing, per meal enhancement of protein has also drawn attention as a specific dietary strategy to optimize protein intake, rather than focusing on total daily protein intake [30,31]. Specific feeding strategies that optimize protein anabolism during intentional energy intake restriction, including the source, timing, distribution and specific amino acid constituents (e.g., leucine), may prevent weight-loss-induced sarcopenia [32]. However, careful medical monitoring and individualized medical nutrition therapy delivered by an Accredited Dietitian is required when optimizing protein intake during intentional energy intake restriction in obese older adults. The challenges associated with weight loss diets in obese older adults are indeed recognized; as such, alternative approaches are also needed to attenuate declines in lean body mass, bone mineral density, strength, and physical function.

The Special Issue in *Healthcare* titled ‘Strategies to Manage Obesity in Older Adults’ was devoted to collecting original research studies investigating therapeutic and alternative treatment strategies (lifestyle, behavioral and pharmacological) for the management of obesity in ageing. Firstly, Youssef et al. [33] compared the efficacy of two training modalities, high-intensity interval training (HIIT) versus moderate intensity continuous training (MICT), on physical function, muscle function, body composition, and blood biomarkers in obese older adults over 12 weeks. The investigators reported similar adherence between both exercise groups; however, changes in various parameters were specific to the type of exercise training. Specifically, MICT was most beneficial for reducing relative gynoid fat mass and increasing lower limb muscle strength, whereas HIIT resulted in greater improvements in physical performance, lean body mass, and skeletal muscle markers of mitochondrial content, fusion, and mitophagy [33].

In overweight older females with pre-diabetes, 12-weeks of leisure-time physical activity (aerobic or resistance training) improved cognitive biomarkers and glycemic control [34]. Specifically, concentrations of cognitive biomarkers, including nerve growth factor, brain-derived neurotrophic factor, and Cathepsin-B, all increased in the aerobic and resistance training groups after 12 weeks, which was not observed in the control group [34].

Lastly, in a cross-sectional analysis of overweight and obese community-dwelling, middle-aged, and older adults with and without type 2 diabetes, Buchanan and Villani [35] reported that adherence to a Mediterranean diet was positively associated with gait speed in the type 2 diabetic sample. Although the authors were unable to rule out residual confounding, these novel results suggest that adherence to healthy dietary patterns, such as the Mediterranean diet, may be a suitable dietary strategy to promote healthy musculoskeletal function in overweight and obese adults with diabetes. 

Overall, all studies included in this Special Issue provide novel evidence and an update of the literature on alternative lifestyle approaches (diet and physical activity) to promote musculoskeletal, metabolic, and cognitive health in overweight and obese older adults. 
